# Associations of genetic variations in *EYA4*, *GRHL2* and *DFNA5* with noise-induced hearing loss in Chinese population: a case- control study

**DOI:** 10.1186/s12940-015-0063-2

**Published:** 2015-09-24

**Authors:** Xuhui Zhang, Yi Liu, Lei Zhang, Zhangping Yang, Luoxian Yang, Xuchu Wang, CaiXia Jiang, Qiang Wang, Yuyong Xia, Yanjuan Chen, Ou Wu, Yimin Zhu

**Affiliations:** Hangzhou Center for Disease Control and Prevention, Hangzhou, 310021 P.R. China; Department of Epidemiology and Biostatistics, Zhejiang University School of Public Health, 388 Yu-Hang-Tang Road, Hangzhou, 310058 Zhejiang P.R. China; Hangzhou Hospital for Prevention and Treatment of Occupational Diseases, Hangzhou, 310014 Zhejiang P.R. China

**Keywords:** Noise exposure, Genetic susceptibility, *EYA4*, *GRHL2*, *DFNA5*, Single-nucleotide polymorphism/SNP

## Abstract

**Background:**

Both environmental and genetic factors are attributable to the incidence of noise-induced hearing loss (NIHL). The purpose of this study was to examine the associations between genetic variations in the *EYA4*, *GRHL2* and *DFNA5* genes and the risk to noise-induced hearing loss (NIHL) in a Chinese population.

**Methods:**

A case–control study was conducted with 476 NIHL workers and 475 normal hearing workers matched with gender, years of noise exposure, and intensity of noise exposure. Twelve tag single-nucleotide polymorphisms (SNP) in the *EYA4*, *GRHL2* and *DFNA5* genes were genotyped using nanofluidic dynamic arrays on the Fluidigm platform. Multiple logistic regression was used to analyze the associations of genetic variations with NIHL adjusted by age, smoking/drinking status, and cumulative noise exposure and their interactions with noise exposure.

**Results:**

The SNPs of rs3777781and rs212769 in the *EYA4* gene were significantly associated with NIHL risk. In rs3777781, comparing with the subjects carrying with TT types, the carriers with AT and AA genotypes had the decreased risk of NIHL (OR = 0.721, 95 % CI = 0.522 - 0.996). In rs212769, the AG and AA carriers had increased NIHL risk (OR = 1.430, 95 % CI = 1.014 - 2.016) compared with the subjects with GG genotype. Rs666026 in the associated *GRHL2* gene and rs2521758 in the *DFNA5* gene were marginally t associated with NIHL (*P* = 0.065 and 0.052, respectively). Rs2521758 and rs212769 had significantly interacted with noise exposure (*P* < 0.05).

**Conclusions:**

Genetic variations in the *EYA4*, *GRHL2* and *DFNA5* genes and their interactions with occupational noise exposure may play an important role in the incidence of NIHL.

## Introduction

Noise is the most widespread environmental pollution that exposed in the occupational and living environment. Regular noise exposure leads to noise-induced hearing loss (NIHL), which is the most common occupational disease [1]. NIHL is a complex disease, resulted from the interaction of environmental and genetic risk factors [2]. Besides noise exposure, smoking, organic solvent exposure, higher blood pressure and cholesterol also increased the risk of NIHL [3]. However, the previous findings indicated that the subjects had different degrees of NIHL risk even if they were exposed under similar noise environment. These findings implicated that the genetic susceptibility and its interaction with environmental factors might play important role in the development of NIHL [4, 5].

Animal experiments have proved that genetics contribute to the incidence of NIHL [6, 7]. In human, the previous genetic studies have demonstrated that variants in CDH23 [8, 9], hOGG1 [10], catalase [11], heat shock protein 70 [12], KCNE1 and KCNQ4 [13] associated with the NIHL risk. Recently, we found that the genetic variants in the PCDH15 gene were associated with NIHL risk and this variant also modified the biological effect induced by occupational-noise exposure [14]. However, the known genetic polymorphisms only explained a small part of the etiology in NIHL.

The *EYA4* and *GRHL2* genes are transcription factors, and associated with composition of Corti [15]. The *EYA4* gene is encoded for the protein of Eyes absent homolog 4 (EYA4). This protein acts through its protein phosphatase activity. It also may be important in eye development, and for continued function of the mature organ of Corti. Previous studies indicated that mutations in the *EYA* gene were associated with postlingual, progressive, autosomal dominant hearing loss at the deafness [16]. *EYA4* localizess to the autosomal dominant non-syndromic hearing loss (NSHL) locus DFNA10 on chromosome 6q23. Several mutations in the *EYA4* had been found to be associated with progressive and hearing loss [17–19]. The Grainyhead like 2 (*GRHL2*) gene is located on chromosome 8q22.3 and it is also a transcription factor cellular promoter 2-like 3. It is highly expressed in epithelial cells lining the cochlear Duct. *GRHL2* plays an important role in epithelial tissues as well as in epithelial cell maintenance [20]. Van Laer et al. [21] found the genetic variants in *GRHL2* gene was associated with age-related hearing impairment. Rs611419 in *GRHL2* was also reported to be related with the risk of NIHL in Chinese population [22].

DFNA5 was firstly discovered to be associated with autosomal dominant hearing loss. A mutation in the *DFNA5* gene was found to associated with autosomal dominant hereditary hearing loss [23]. DFNA5 contains two domains separated by a hinge region. The first domain in *DFNA5* induces apoptosis when transfected into cell lines while the second domain may shield the apoptotic-inducing sequences residing in the first domain. Therefore, it is hypothesized that the mutations in *DFNA5* might function on both of sensorineural hearing loss and carcinogenesis through activating the apoptosis [24].

Given that the important roles of *EYA4*, *GRHL2* and *DFNA5* in auditory system, we assumed that the genetic variants in these three genes might associate with the risk of NIHL. To examine this hypothesis, we genotyped 12 tagSNPs in the *EYA4*, *GRHL2* and *DFNA5* genes in 476 NIHL workers and 475 normal hearing workers, and analysised the associations of these SNPs with NIHL. We also explored the interaction effects among these SNPs and noise exposure.

## Methods

### Subjects

The design of this study were previously described in detail [14]. Briefly, subjects in this study included 476 NIHL workers and 475 normal hearing workers. All the subjects were recruited from a cross-sectional survey of 4419 occupational noise- exposed workers conducted between March 1, 2011 and December 31, 2012. In this survey, the workers were employed in the noise- exposed factories of mechanical equipment and household appliance manufacturing, steel construction, and cigarette production/packaging in Hangzhou city, Zhejiang province, China. Intensity of noise in the workplace was determined by a noise statistical analyzer (AWA6218; Westernization Instrument Technology Co., Ltd., Beijing, China). Noise exposure was evaluated with equivalent continuous dB (A)- weighted sound pressure levels (L_EX,8h_) according to Occupational Health Standard of the People’s Republic of China: Measurement of Noise in the Workplace (GBZ/T 189.8-2007) (China, 2007). All the subjects received annual health examinations, including routine physical examination, pure tone audiometry (PTA), epidemiological investigation, and whole-blood collection. The inclusion criteria of subjects in this cross-sectional survey were as follows: (1) Working at noised exposed workplace and L_EX,8h_ was >85 dB (A); (2) Cumulative time of noise exposure of >1 year. Cumulative time of noise exposure of each worker was recorded according to the files of occupational health surveillance and verified with epidemiological interview; (3) Han ethnicity. The subjects were excluded if they had a family history of hearing loss or histories of the diseases such as otitis, other otological diseases, head injury, exposure to explosives, or ototoxic drug administration. The study protocol was approved by the Research Ethics Committees of Hangzhou Center for Disease Prevention and Control, Zhejiang, China.

### PTA and NIHL assessment

After participants stopped noise exposure for >12 h, audiometry was carried out for each subject using a Madsen Voyager 522 audiometer (Madsen, Taastrup, Denmark) in a soundproof room with a background noise level of <25 dB (A). Hearing thresholds of both ears were determined with the ascending method in 5-dB steps at frequencies of 500, 1000, 2000, 3000, 4000, and 6000 Hz. All the evaluations were performed by trained physicians using standard procedures. In order to exclude the confounding effects by age and gender, the audiometric raw data were calibrated for the effects of age and gender on the basis of the Diagnostic Criteria of Occupational NIHL (Chinese National Criteria GBZ49-2007). The hearing threshold at high frequency (HTHF) by PTA was defined as the average at 3000, 4000, and 6000 Hz for each ear, and the hearing threshold at speech frequency (HTSF) was defined as the average at 500, 1000, and 2000 Hz for each ear.

### Physical examination and epidemiological investigation

A physical examination was performed for each subject. Parameters such as height, weight, systolic and diastolic blood pressure levels were measured by trained physicians following a standard protocol. Face-to-face interview was used to collect epidemiological data using a structured questionnaire administered by trained professional physicians. The information in the questionnaire included demographic characteristics; smoking/drinking status; history of medical conditions and drug use; history of exposure to noise, vibration, and toxic chemicals in the workplace; health habits; use of ear protection for noise.

### Definitions of NIHL and control subjects

NIHL group included the workers with normal hearing before exposure, >1 year of occupational noise exposure, and an HTHF >40 dB of hearing level (HL). In order to exclude hearing loss induced by non-noise exposure, the workers were excluded from the study if their differences of HTHF between left and right ears were great then 35 dB (HL). The normal group included the workers with >1 year of occupational noise exposure, and hearing thresholds <25 dB (HL) at each frequency. In order to control the environmental confounders, control subjects were individually matched with NIHL subjects according to the variables of gender, intensity of noise and years of noise exposure. Because the majority of the subjects in the cross-sectional study were males (about 91.7 %), the subjects in this study were restricted on males.

### SNP selection and genotyping

Tag SNPs in the *EYA4、DFNA5、GRHL2 genes* were selected as tSNPs on the basis of the HapMap database and previous findings from the literature. The criteria for searching for tSNPs were as follows: MAF (minor allele frequency) of CHB > 0.10, and a linkage disequilibrium value of r^2^ >0.8. Twelve candidate SNPs were selected using these criteria: rs2521758, rs2521768 in the *DFNA5* gene, rs3777781, rs212769, rs3777849, rs465147, rs3777860 in the *EYA4* gene, rs666026, rs471757, rs10955255, rs682769 and rs1981361 in the GRHL2 gene.

Whole-blood and serum samples were collected from each subject after an overnight fast. Genomic DNA was extracted from peripheral blood using the Toyobo MagExtractor Genomic DNA Purification Kit (Toyobo, Osaka, Japan) following the manufacturer’s protocol. All the subjects were genotyped using nanofluidic dynamic arrays on the Fluidigm platform (South San Francisco, CA, USA) in Bio-X Institutes (Shanghai, China) [25]. Repeated control samples were set in every genotyping plate, and the concordance was >99 %.

### Statistics

Cumulative noise exposure (CNE) was calculated as CNE = 10 × log(10^SPL^ × years of noise exposure), where SPL means the sound pressure level [dB (A)] of noise exposure. Continuous variables for the normal distribution were expressed as mean ± standard deviation (SD) and as median (P25, P75) for skewed distribution. Categorical variables were expressed as frequencies (%). Student’s *t* test was used to examine the statistical significance for continuous variables, and the *χ*^2^ test was used for categorical variables. Hardy-Weinberg equilibrium were tested using Pearson’s *χ*^2^ for each SNP among control subjects, and the SNPs with deviating from Hardy–Weinberg equilibrium were excluded from the analysis. Multiple logistic regression was used to calculate the OR and 95 % CI, and gene–environmental interactions modified by confounders such as age, smoking/drinking status, and Cumulative noise exposure (CNE). All statistical analyses were performed using SPSS 19.0 for Windows (IBM Corporation, Armonk, NY, US).

## Results

### Basic characteristics of the subjects

The basic characteristics of the subjects have been described in detail in our previous study [14]. Briefly, the subjects included 476 NIHL subjects and 475 control subjects. All the subjects were males. The mean age was 36.6 ± 8.5 years in NIHL subjects, which was significant elder than that in control subjects (32.8 ± 8.0) (*P* < 0.001). No significant differences were found between NIHL and normal hearing groups in the distributions of smoking and drinking status, years of noise exposure, median of noise intensities (*P* > 0.05). The median of cumulative noise exposure (CNE) in the NIHL group [95.5 (91.5, 100.5)] was a little, but not significantly higher than that in the normal hearing group [94.3 (91.0, 97.8)] (*P* > 0.05).

### Associations of EYA4, GRHL2 and DFNA5variants with the risks of NIHL

Basic information of SNPs in these three genes and the significance are shown in Table 1. Nine of studied SNPs in controls were in Hardy–Weinberg equilibrium distribution (*P >* 0.05), except for rs3777849, rs471757 and rs682769, which were excluded in the analysis.

**Table 1:** Distributions of allele and genotype in the subjects of NIHLand normal hearing groups

gene	SNP	A1/A2	maf	case^a^	control^a^
*EYA4*	rs3777781	A/T	0.430	72/235/166	98/236/137
	rs212769	A/G	0.141	8/129/336	5/112/354
	rs3777849^#^	A/G	0.353	70/175/228	85/182/204
	rs465147	T/C	0.010	0/7/465	0/11/459
	rs3777860	A/G	0.355	62/217/194	57/215/200
*GRHL2*	rs666026	G/T	0.298	46/204/222	40/186/244
	rs471757^#^	T/C	0.431	89/217/167	105/208/158
	rs10955255	G/A	0.220	16/164/292	23/173/275
	rs682769^#^	A/G	0.191	10/155/308	11/163/297
	rs1981361	A/G	0.289	36/200/237	34/205/232
*DFNA5*	rs2521758	G/T	0.019	0/14/458	1/20/450
	rs2521768	C/T	0.209	19/148/305	27/155/289

^a^Presented as the order of A1A1/A1A2/A2A2. A1: the minor allele; A2: the major allele

^#^ *P* values < 0.05 after Hardy-Weinberg equilibrium tests

Table 2 shows the odds ratios (ORs) of genotypes in the associated SNPs. The frequency of AT/AA in rs3777781 in NIHL group was 64.8 %, which was significantly lower than that in control group (70.8 %) (*P* < 0.05). Comparing with the subjects with homozygotes of wild type (TT), the subjects carrying with variant A allele (AT and AA) decreased the risk of NIHL with the OR of 0.721 (95 % CI = 0.522 - 0.996). In rs212769, the percentage of the genotypes with AG and AA was 29.0 % in the NIHL group and 24.8 % in control group (*P* < 0.05). The subjects carrying with A allele (AG or AA) increased the risk of NIHL with an adjusted OR of 1.430 (95 % CI = 1.014 - 2.016) comparing with the subjects with homozygous wildtype (GG). We also found that genotypes of rs666026 and rs2521758 had the marginal associations with NIHL (*P* = 0.065 and 0.052, respectively).

**Table 2:** Associations of candidate SNPs with the risk of NIHL

Gene	SNP	Genotype	Control n (%)	Case n (%)	P-value	OR (95 % CI)^a^
*EYA4*	rs3777781	TT	137 (29.2)	166 (35.2)		1
		AT	236 (50.3)	235 (49.8)	0.116	0.759 (0.539, 1.070)
		AA	96 (20.5)	71 (15.0)	0.015	0.570 (0.363, 0.895)
		P_trend_			0.019	
		AT/AA	332 (70.8)	306 (64.8)	0.047	0.721 (0.522, 0.996)
	rs212769	GG	354 (75.2)	336 (71.0)		1
		AG	112 (23.8)	129 (27.3)	0.062	1.395 (0.984,1.979)
		AA	5 (1.1)	8 (1.7)	0.214	2.369 (0.608,9.234)
		P_trend_			0.033	
		AG/AA	117 (24.8)	137 (29.0)	0.041	1.430 (1.014,2.016)
*GRHL2*	rs666026	TT	244 (51.9)	222 (47.0)		1
		GT	186 (39.6)	204 (43.2)	0.118	1.287 (0.938,1.767)
		GG	40 (8.5)	46 (9.7)	0.121	1.554 (0.891,2.71)
		P_trend_			0.051	
		GT/GG	226 (48.1)	250 (53.0)	0.065	1.329 (0.983,1.798)
*DFNA5*	rs2521768	TT	289 (61.4)	305 (64.6)	0.158	1
		CT	155 (32.9)	148 (31.4)	0.249	0.825 (0.595,1.144)
		CC	27 (5.7)	19 (4.0)	0.089	0.524 (0.249,1.104)
		P_trend_			0.065	
		CT/CC	182 (38.6)	167 (35.4)	0.124	0.782 (0.571, 1.070)
	rs2521758	TT	450 (95.5)	458 (97.0)		1
		GT	20 (4.2)	14 (3.0)	0.078	0.474 (0.206,1.088)
		GG	1 (0.2)	0 (0.0)	1.000	
		GT/GG	21 (4.5)	14 (3.0)	0.052	0.441 (0.194,1.006)

^a^calculated with logistic regression adjusted by age, CNE, smoking, drinking

### Interaction and stratification analysis of EYA4, GRHL2 and DFNA5 by noise intensity and CNE

After adjusted by age, drinking, smoking status, significant multiplicative interactions for NIHL were found between rs2521758 and CNE (*P* = 0.040, OR_int_ = 0.794, 95 % CI = 0.638-0.989), and between rs212769 and noise intensity (*P* = 0.041, OR_int_ = 1.100, 95 % CI = 1.004 - 1.205) (Table 3). In the noise exposure with CNE < 95, the subjects with the genotypes of GT and GG in rs2521758 were found to have the decreased NIHL risk (OR = 0.115, 95 % CI = 0.014 - 0.921). However, no significant association of rs2521758 was found in the noise exposure with CNE >95 (*P* > 0.05). The subjects carrying A allele (AG or AA) in rs212769 had significantly increased the risk of NIHL in the noise intensity of ≥ 88 dB(A) with the OR of 1.727 (95 % CI = 1.009-2.954), not significantly in the noise intensity of < 88 dB(A).

**Table 3:** Stratified analysis of associated SNPs by CNE and intensity of noise exposure

Exposure	SNP	Genotype	Control	Case	P1	P2	OR (95 % CI)
CNE							
<95	rs2521758	TT	244	209	0.040		1
		GT/GG	12	3		0.042^a^	0.115 (0.014, 0.921)^a^
≥95		TT	205	232			1
		GT/GG	9	10		0.587^a^	0.761 (0.284, 2.042)^a^
Intensity (dB)							
<88	rs212769	GG	211	181	0.041		1
		AG/AA	72	71		0.503^b^	1.169 (0.740, 1.849)^b^
≥88		GG	143	155			1
		AG/AA	45	66		0.046^b^	1.727 (1.009, 2.954)^b^

P1: *P* value of multiplicative interaction, which calculated by logistic model Multiplicative term; P2: *P* value of the association in each stratum

^a^calculated with logistic regression adjusted by age, smoking, drinking

^b^calculated with logistic regression adjusted by age, smoking, drinking, years to noise exposure

## Discussion

This study examined the associations of 12 SNPs in *EYA4*, *GRHL2* and *DFNA5* genes with the risk of NIHL. We found that the genetic variations of rs3777781, rs212769, rs666026 and rs2521758 were associated with NIHL risk. Rs2521758 and rs212769 had interacted with noise exposure. These findings suggested that genetic polymorphisms in *EYA4*, *GRHL2* and *DFNA5* and their interactions with noise exposure might play important roles in NIHL incidence.

Konings et al. [26] had demonstrated that the SNPs in the *EYA4* gene were associated with NIHL risk in Sweden and Poland populations. In this study, we investigated four SNPs in the *EYA4* gene and replicated that rs3777781 and rs212769 were associated with NIHL risk in Chinese population. Furthermore, interaction effect was found between genotype of rs212769 and intensity of noise exposure. After stratification, significant association was only found in the intensity of noise ≥88 dB and non significance in the intensity of noise <88 dB. The combined effect of rs212769 and intensity of noise was 1.710 (1.115 – 2.623). To our knowledge, this is the first study to investigate the association of SNPs in *EYA4* gene in Chinese population. In European, two intronic SNPs in the *EYA4* gene was found to be significantly associated with age-related hearing impairment (ARHI). Rs212765 had reached the significance of 0.000565 with ARHI [21]. Although the biological mechanism is unclear, these findings suggested that genetic variation may be associated with the NIHL risk. EYA4 is a transcriptional activator and plays an important in eye development, and for continued function of the mature organ of Corti. Previous studies mainly focused on the mutations in *EYA4* on hearing loss and had found that several causative mutations caused non-syndromic hearing loss [16–18, 20]. Together the evidences of present study with Kongs’ study, it indicated that genetic variation in *EYA* gene might be a biomarker of genetic susceptibility for NIHL.

*GRHL2* is known as a transcription factor cellular promoter 2-like 3 and highly expressed in epithelial cells lining the cochlear duct. It acts a critical role in embryonic development and maintains epithelial cells throughout life [20]. Animal study indicated *GRHL2* involved in otic development and hearing. The mutation in this gene associated with hearing loss in humans. As a transcription activator, *GRHL2* regulates apical junctional proteins and desmosomal cadherin expression. Peters et al. firstly reported that mutation in *GRHL2* could induce *DFNA28* [20]. In European, genetic variation of *GRHL2* associated with ARHI. Rs10955255 had arrived the significance of 8.38 × 10^−5^ with ARHI [21]. For genetic susceptibility of NIHL, rs1981361 in Sweden and rs682769 in Poland population were found to be associated with NIHL [11]. However, we didn’t observe the associations of these SNPs in the present study. We found marginal significance of rs666026 (*P* = 0.051), which was no significance in Sweden and Poland populations . Li et al. also found the significant association of rs611419 in Chinese population and the AT/TT genotypes conferred decreased NIHL risk [22]. These results might reflect the race difference in genetic susceptibility.

*DFNA5* was first discovered in an extended Dutch family with autosomal dominant nonsyndromic hereditary HL [27, 28]. Four mutations in introns 7 or 8 of *DFNA5* have been reported to cause HL [29]. These four mutations lead to skipping of exon 8, resulting in a frameshift, and presumably causing premature truncation of the DFNA5 protein. It was suggested that *DFNA5*-associated HL represents a mechanism of gain-of-function. DFNA5 contains two domains separated by a hinge region. The first domain in DFNA5 induces apoptosis when transfected into cell lines while the second domain may shield the apoptotic- inducing sequences residing in the first domain. It is a well known that apoptosis contributes to several acquired forms of hearing impairment. Apoptosis is a key contributor to the development of presbycusis, age-related hearing loss and cancer [24]. Apoptosis contributes to the pathology of acquired hearing impairment and to genetic hearing impairment [30]. The mutation in DFNA5 leads to a type of hearing loss that closely resembles the frequently observed age related hearing impairment (ARHI). However, no significant associations of four coding SNPs in *GRHL2* gene were found to be related with ARHI [31]. Rs2521772 associated with NIHL in Sweden population, however, no significance in Poland population. In this study, we found the genotypes of rs2521758 had significant association with NIHL although its MAF is only 0.019. These results indicated that genetic variation in *GRHL2* gene might associated with NIHL risk.

This study was carried out based on a cross-sectional study on occupational noise-exposed worker. We recruited NIHL cases and sex, intensity of noise and years of noise exposure matched healthy controls under similar environmental exposure. Therefore, there were high comparability between NIHL cases and controls. We have also excluded the subjects whose audio-impairment were caused by risk factors of non-occupational exposure. The difference between two groups reflected the potential effect of genetic susceptibility. However, there were some limitations in this study. Due to the majority of the noise-exposed workers were males, therefore, the subjects in this study were also restricted to males. Although the sample size was relatively large in the previous studies, however, due to the lower biological effects of an individual SNP, the power of statistics might not be enough. On the other hand, the significance could not arrived at the multiple correction. In order to decrease the false positive and negative errors, the results should be validated in other independent population with large sample size including females.

## Conclusion

We found that SNPs of rs3777781, rs212769, rs666026 and rs2521758 were associated with NIHL risk. Interaction effects were found between rs2521758, rs212769 and noise exposure. These findings suggested that genetic variations in *EYA4*, *GRHL2* and *DFNA5* and their interactions with noise exposure may play important roles in NIHL incidence.

## Abbreviations

SNP: Single-nucleotide polymorphism
CI: Confidence interval
NIHL: Noise- induced hearing loss
OR: Odds ratio
MAF: Minor allele frequency
CHB: Han Chinese individuals from Beijing
CNE: Cumulative noise exposure
